# CNN-Based Classification of Optically Critical Cutting Tools with Complex Geometry: New Insights for CNN-Based Classification Tasks

**DOI:** 10.3390/s25051575

**Published:** 2025-03-04

**Authors:** Mühenad Bilal, Ranadheer Podishetti, Tangirala Sri Girish, Daniel Grossmann, Markus Bregulla

**Affiliations:** Application Cluster “Digital Production” Progarm, AImotion Bavaria Instiutute, Technische Hochschule Ingolstadt (THI), Esplanade 10, 85049 Ingolstadt, Germany; ranadheer.podishetti@thi.de (R.P.); srigirish.tangirala@thi.de (T.S.G.); daniel.grossmann@thi.de (D.G.); markus.bregulla@thi.de (M.B.)

**Keywords:** tool classification, machining tools: Grad-CAM CNN, ResNet50, training performance, neural network performance, sustainability, ResNet152

## Abstract

Sustainability has increasingly emphasized the importance of recycling and repairing materials. Cutting tools, such as milling cutters and drills, play a crucial role due to the high demands placed on products used in CNC machining. As a result, the repair and regrinding of these tools have become more essential. The geometric differences among machining tools determine their specific applications: twist drills have spiral flutes and pointed cutting edges designed for drilling, while end mills feature multiple sharp edges around the shank, making them suitable for milling. Taps and form cutters exhibit unique geometries and cutting-edge shapes, enabling the creation of complex profiles. However, measuring and classifying these tools for repair or regrinding is challenging due to their optical properties and coatings. This research investigates how lighting conditions affect the classification of tools for regrinding, addressing the shortage of skilled workers and the increasing need for automation. This paper compares different training strategies on two unique tool-specific datasets, each containing 36 distinct tools recorded under two lighting conditions—direct diffuse ring lighting and normal daylight. Furthermore, Grad-CAM heatmap analysis provides new insights into relevant classification features.

## 1. Introduction

The importance of repairing equipment, tools, and recycling waste materials is growing in Germany and worldwide, particularly in the context of sustainability and supply chain challenges. Tool manufacturers and grinding factories, such as Linner GmbH Werkzeugfabrik, process over 1,200 tools daily for various applications, each with distinct performance characteristics and usage intervals. Given the complex geometry of these tools, each one must be accurately measured and identified according to its technical parameters. This measurement is essential for programming the CNC machine’s regrinding profile. Until now, this process has primarily been performed manually. However, through the Digital Tool Intelligence (DTL) cooperation project, Linner GmbH Werkzeugfabrik aims to enhance this process (website: https://herionlinner.com/linner-gmbh-werkzeugfabrik/ (accessed on 2 February 2025)) and intends to automate this process using convolutional neural networks (CNNs). Image classification is a well-established research topic that has garnered significant attention in recent years. It is a fundamental aspect of computer vision and serves as the basis for various visual recognition tasks. The effectiveness of classification networks is crucial for the advancement of AI-based applications. Typically, factors such as image quality, network architecture, and dataset complexity play a vital role in determining model performance. Various applications have been successfully tested to enhance the performance of classification networks [1]. These application areas include object tracking [2], super-resolution technology [3], object recognition [4], segmentation [5], and video classification [6].

In AI-based applications for computer vision in the field of cutting tool inspection, significant advancements have been made in quality control. Researchers are developing image-processing applications for detecting wear on milling tools. Various lighting techniques have been employed, including circular ring arrays of light-emitting diodes (LEDs) [7], direct diffuse illumination [8], and dome illumination [9]. New approaches using advanced techniques have demonstrated that illumination plays a significant role [10,11,12,13]. These These methods provide high illumination intensity, reduce shadow effects, enhance the visibility of relevant features, and are easy to install on different devices.

Diffuse lighting often detects optical damage on reflective surfaces but cannot completely eliminate unwanted reflections. These undesirable light artifacts arise from the varying curvatures and orientations of the tool’s surface, resulting in both regular and specular reflections. The effect of diffuse lighting on the classification performance of a specific dataset has not been thoroughly explored. This paper offers a comprehensive analysis of how lighting impacts cutting tool classification and presents insights into the performance of two well-established neural network architectures: ResNet 50 and its extended version, ResNet 152. A unique dataset has been utilized to evaluate different training strategies, including training from scratch, freezing weights, and transfer learning. These insights are of particular importance for establishing AI-based classifications of tools in the industrial environment and for selecting the right grinding profile for regrinding the tools, which would otherwise only be possible with the extensive specialist knowledge required for programming the CNC machines.

## 2. State of the Art

### 2.1. CNN Architectures and Datasets

There has been a significant development in the field of CNN (convolutional neural network)-based classification in recent decades. As early as 1998, Yann LeCun et al. introduced the CNN architecture—a milestone in image classification and object recognition [14]. It became LeNet-5 and paved the way for many later developments. Among the first practical applications were face recognition and medical image processing. A few decades later, growth has accelerated massively. Another milestone in developing AI-supported classification followed in 2012: Krizhevsky et al. developed AlexNet. This deep CNN architecture won the ImageNet Large Scale Visual Recognition Challenge (ILSVRC) by a large margin [15]. Its success was mainly due to the use of GPUs for processing, which made it possible to train and optimize deep neural networks practically. The VGG (Visual Geometry Group) architecture was introduced in 2014, a deep CNN architecture with 16 layers using simple 3 × 3 convolutional filters [16]. Although VGG-16 performed convincingly on the ILSVRC dataset with an accuracy of 71.5%, it had a high computational cost. The CNN architectures *AlexNet* [15] and *VGGNet* [16] established a foundation for deeper networks capable of tackling more complex image recognition tasks. VGGNet, in particular, focused on using smaller convolutional filters to enhance feature extraction.

However, as neural networks became deeper, problems such as gradient descent arose that made training more difficult. This led to the introduction of the ResNet architecture (Residual Network) by Kaiming He et al. in 2015 [17]. The skip connections in ResNet enabled a better gradient flow, making very deep networks trainable. The ResNet-50 and ResNet-152 variants achieved accuracy on the ILSVRC dataset of 76.6% and 78.3%, respectively. ResNet, introduced by He et al. in 2015 [18], represented a significant breakthrough in deep learning due to its residual connections, which help mitigate the vanishing gradient problem. This architecture allows for considerably deeper networks—up to hundreds of layers—without sacrificing performance. The introduction of ResNet marked a shift toward deeper architectures for classification tasks, making it an ideal choice for applications involving complex tool shapes, where capturing intricate details is essential.

Further progress was made with DenseNet-161, developed by Gao Huang et al. [19]. It introduced the concept of dense connections, where each layer is connected to all previous layers. As a result, the feature reuse approach was introduced, meaning fewer filters were needed per layer. For example, DenseNet-121 required only 8 million parameters, while ResNet-50 had around 25 million and ResNet-152 around 60 million. Besides reducing training parameters, DenseNet-121 showed better generalization on smaller datasets. In 2019, further significant progress was made with introducing the EfficientNet architecture by Mingxing Tan et al. [20] in 2019. It utilized optimized scaling of depth, width and resolution and introduced the Mobile Inverted Bottleneck Convolution (MBConv) blocks of MobileNetV2. EfficientNet, therefore, required less computing power than previous CNNs. Different variants were developed, from EfficientNet-B0 (5.3 million parameters) to EfficientNet-B7 (66 million parameters). On the ImageNet dataset, EfficientNet-B7 achieved an accuracy of 84.3%, while EfficientNet-B0 achieved 77.1%. In 2021, the research team led by Li et al. presented an extended version called EfficientNet-X. This architecture achieved an accuracy 86% on ImageNet with an even more efficient computational strategy—with 30% fewer FLOPs than EfficientNet-B7.

However, despite the success of CNN-based architectures, newer architectures such as Vision Transformers (ViTs) [21] have demonstrated promising results in reducing computational complexity while maintaining or improving accuracy. Meanwhile, ViTs move away from traditional convolutional neural network (CNN) structures by utilizing self-attention mechanisms, allowing them to process images in a more global context. New types of datasets are of crucial importance for the validation of neural networks. In 1998, the MNIST dataset was published as the first significant dataset for validating neural networks in classification tasks. It comprises 60,000 training images and 10,000 test images of handwritten digits (0–9), each with a resolution of 28 × 28 pixels. MNIST is considered one of the first and most widely used base datasets for simple classification tasks [14]. Another important dataset for classification tasks is CIFAR-10 and CIFAR-100. CIFAR-10 consists of 60,000 color images with a size of 32 × 32 pixels, which are divided into 10 classes, including vehicles such as cars and natural objects such as animals. With 100 classes, CIFAR-100 is an extended version and is often used as a benchmark for smaller image datasets [22].

With the progress of artificial intelligence and the development of ever more powerful neural networks, the need for larger and more complex datasets has also grown. ImageNet, published in 2010, is one of the most important image datasets for training deep neural networks. It comprises over 1000 different classes and was instrumental in the development of powerful models such as AlexNet and ResNet, which were used in the ImageNet Large Scale Visual Recognition Challenge (ILSVRC) [23]. In addition to the classic image datasets, alternative versions were developed for specific use cases. Fashion-MNIST, an extension of MNIST, was published as an alternative benchmark for the image classification of fashion items. The dataset contains 70,000 images with a resolution of 28 × 28 pixels and covers 10 categories, including shoes, pants, and jackets [24]. Another significant addition is Tiny ImageNet, which was launched in 2017 as a scaled-down version of ImageNet. It contains 100,000 images with a resolution of 64 × 64 pixels and is used in particular for evaluating models on smaller datasets [25]. Specific datasets for image classification tasks have also been developed in remote sensing and play a central role in modern research. In particular, they have been used to validate ResNet50 and ResNet152. One example is the So2Sat dataset, a multispectral satellite dataset with over 400,673 images used for urban structure classification. In a study by Ivica Dimitrovski [26], ResNet50 achieved a moderate accuracy of 59.587% on this dataset, while ResNet152 achieved an accuracy of 61.477%. The application of deep learning architectures to classify tools has so far only been studied to a limited extent and for only five tool types [27]. To the best of our knowledge, we hereby present a unique dataset comprising tools of different types with visually critical features. This consists of 36 classes and is used to evaluate ResNet50 and ResNet152 to validate the performance of these models on a domain-specific dataset. In addition, different training strategies are investigated to demonstrate the potential of a CNN-based application for tool classification and maintenance.

### 2.2. Transfer Learning and Pre-Training

Pre-trained models have become a standard method for enhancing the performance of deep learning applications in specialized domains with limited data. Models such as ResNet, EfficientNet, and Vision Transformers (ViTs) that are pre-trained on large datasets like ImageNet can transfer general features learned from millions of images to new tasks [28]. In this work, we addressed the question: Can ResNet architectures, particularly with pre-trained weights, also perform well on our specific dataset? These pre-trained models were essential for managing the complexity of the tool dataset, enabling the network to generalize more effectively even with a smaller amount of training data. Recent studies indicate that freezing the pre-trained weights in the early layers of convolutional neural networks (CNNs), as demonstrated in this work, helps preserve the general features extracted by the pre-trained model [29]. In contrast, fine-tuning allows for greater adaptability but carries the risk of overfitting, especially when working with a small or highly specific dataset [30]. These findings are consistent with our results, where freezing the pre-trained weights outperformed fine-tuning strategies on the tool dataset, despite the specificity of the datasets.

### 2.3. Industrial Applications of Deep Learning

In industrial settings, convolutional neural networks (CNNs) have been utilized for various tasks such as object detection [4], defect detection [8], and wear detection on milling tools [7]. These applications often require robust models capable of handling variations in lighting, surface reflectivity, and tool geometry. Machine vision systems that utilize image processing techniques, such as dome illumination and direct diffuse illumination, have been explored for quality control [9]. However, few studies have focused on tool classification [31], making our work a novel contribution to this field.

While most research in this area has concentrated on detecting tool wear or surface defects, our study adopts a different approach [32]. This work focuses on classifying tools based on their geometry and coating, which significantly contributes to the existing literature. Previous studies, such as those by Wu et al. [7] and Wei et al. [9], have demonstrated that employing CNNs for wear detection can significantly reduce manual inspection times. Another computer vision-based wear detection and analysis approach has shown that proper illumination can dramatically improve the results [10,11,12,13]. However, these systems are typically limited to specific tool types and conditions.

## 3. Methodology

### 3.1. Lighting Behavior of Milling Tools

Our dataset includes tools with both cylindrical and complex shapes. These tools exhibit a high reflection coefficient due to their coatings, such as titanium nitride (TiN). The Phong reflection model effectively describes this behavior.

**Figure 1:**
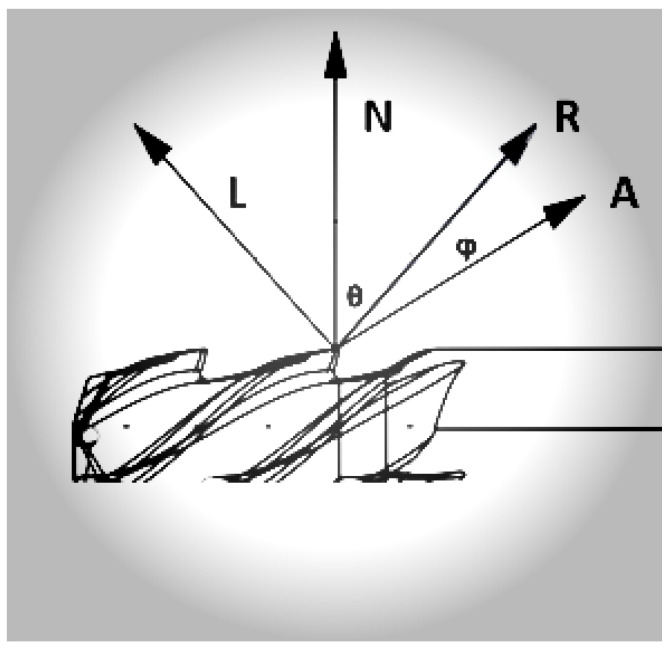
The Phong model is used to simulate the reflection behavior of light. The specular component ’R’ results in a specular highlight, which depends on the orientation of the surface of the tool relative to the observer (A); normal vector (N); and point light source (L), with R representing the unit vector directed towards the ideal specular reflection, θ representing viewing angle relative to the specular reflection direction R, and finally, ϕ representing the angle made by L and R with N.

The Phong reflection model explains how the perceived brightness of an object’s detected light intensity (II) is composed of three components: ambient, diffuse, and specular light [33]. The total detected intensity (I) can be expressed as follows:

(1) $$I=I_{a}K_{a}+I_{l}K_{a}\cos\theta +I_{l}K_{d}\cos\theta +I_{l}K_{s}\cos\alpha$$

where $K_{a}$, $K_{d}$ and $K_{s}$ are the ambient, diffuse, and specular light coefficients, respectively. $I_{a}$ and $I_{l}$ are the intensities of ambient light and direct light from the light source, respectively. θ is the angle between the incident light and the normal on the object’s surface. α is the angle between the reflected light and the line of sight, and *n* is the specular reflection coefficient. The schematic representation of the light dispersion on the tool surfaces is shown in Figure 1.

Our research is the first to explore tool classification, addressing a gap in the current literature. For this paper, a dataset that includes various types of tools under two different lighting conditions has been created. We aim to apply this analysis to tool maintenance with a focus on sustainability. By classifying tools, we can assign categories and develop regrind profiles for each tool type, which could potentially revolutionize tool maintenance practices. Additionally, this work investigates the impact of different lighting conditions on model performance by employing various training strategies.

### 3.2. CNN Architecture

This paper focuses on ResNet architectures, specifically ResNet50 and ResNet152, which are well-established models renowned for their reliability in image classification tasks. One of the main advantages of ResNet is its capability to manage deeper networks without facing challenges such as vanishing gradients, thanks to the implementation of residual connections. This feature makes ResNet an excellent choice for our initial experiments with this new dataset.

**Figure 2:**
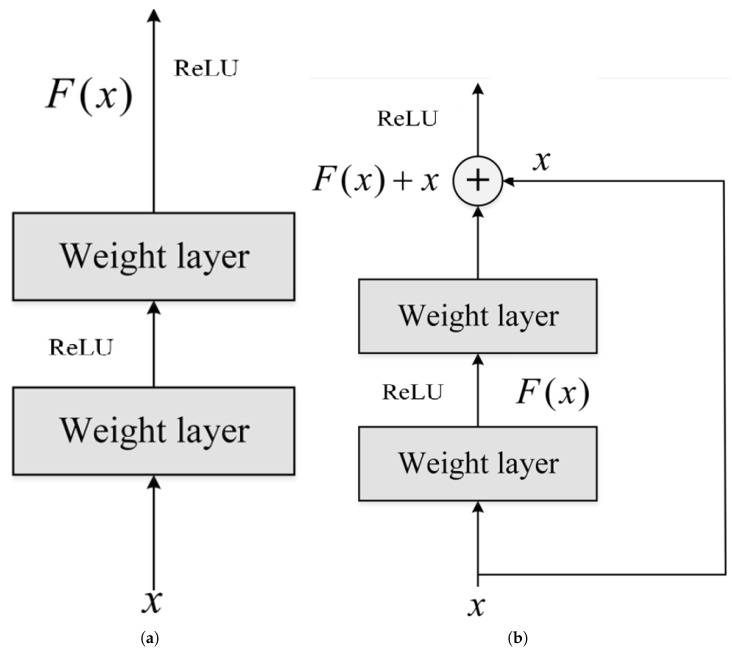
Comparison of both training processes according to different architectures. (**a**) Training in usual CNN architecture. (**b**) Training in ResNet architecture.

Figure 2 illustrates the concept of residual learning. In a traditional neural network, the layers learn a function H(x) based on the input signal *x*. In a ResNet, this function is reformulated into a residual block.

Instead of learning H(x) directly, each layer learns a residual function F(x) so that:

H(x)=F(x)+x

Here, *x* is the input to the layer, and F(x) is the residual function to be learned. The aim is to learn F(x) in such a way that the shift output H(x) is obtained by adding the input *x* and the residual function F(x). This avoids the disappearance of the gradient, which is often a problem with deep neural networks and multiple classes.

**Figure 3:**
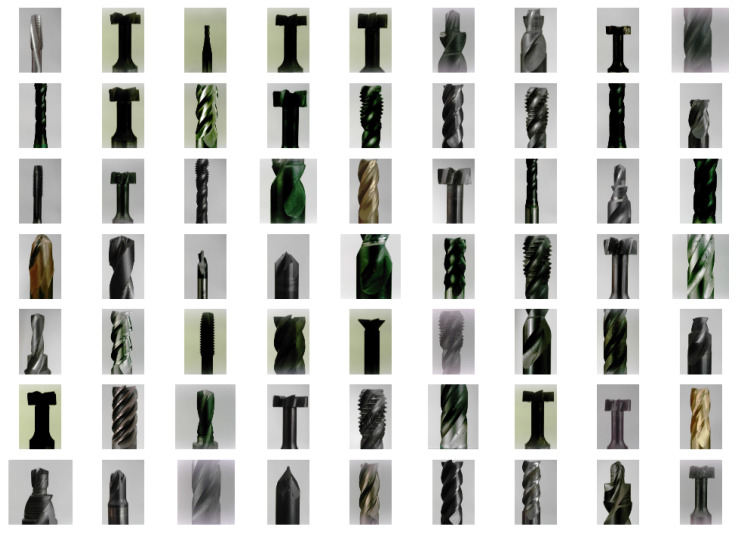
Image grid showing images of distinct tools in the dataset acquired using daylight lighting.

**Figure 4:**
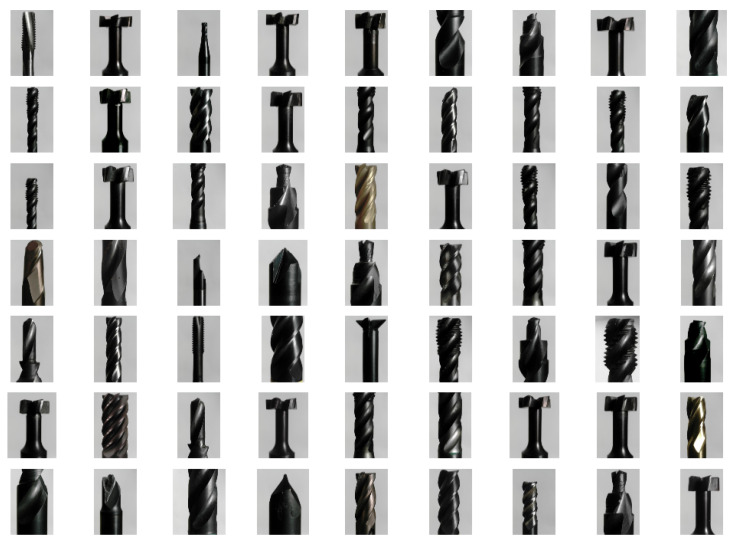
Image grid showing images of distinct tools in the dataset acquired using diffused ring lighting.

**Figure 5:**
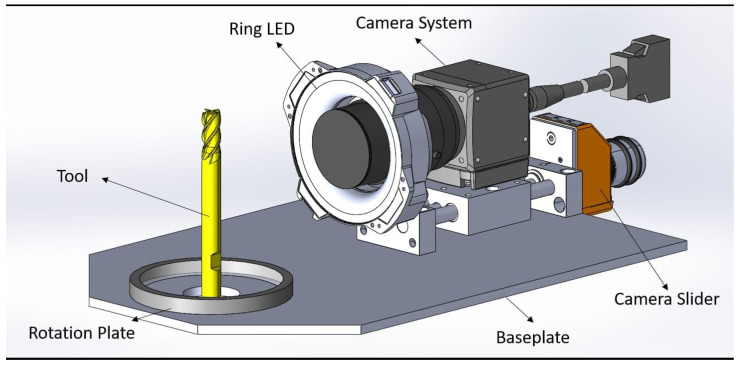
Experimental setup for tool imaging, featuring a rotation plate for tool positioning, a ring LED for uniform illumination, a camera system mounted on a slider for precise adjustments, and a stable baseplate for system stability and reproducibility.

A comprehensive dataset was compiled, comprising images sized 448 × 448 pixels captured under two lighting conditions: natural daylight (Figure 3) and diffused ring light (Figure 4). The acquisition system is illustrated in Figure 5. An ArduCam Mini OV5642, with a resolution of 2592 × 1944, was used to capture the images. The dataset includes 36 different milling tools varying in geometry, coating, and size. For each of the 36 tools, 24 images were captured, resulting in a total of 1.728 images for both lighting conditions. The images were taken from the same angles while the tool was rotated at equal angular intervals. The dataset was then divided into training, validation, and test sets in a 60:20:20 split, ensuring a balanced representation of all classes. Images taken with diffuse ring lighting had an irradiation angle of 0 degrees. This means that the tools were diffusely illuminated frontally. The daylight images were taken in a room at the same time in daylight.

The uniqueness and specificity of the dataset arise from the various shapes and coatings of the tools, such as:

- Drills;
- End mills;
- Chamfer;
- Surface grinding;
- Multidrill;
- Step drill;
- Twist drill;
- Countersink.

### 3.3. Training Strategy

This paper aims to validate different models trained with different training strategies against two datasets of 36 different classes. Each dataset consists of 864 images, 24 images per tool. The selection of the training strategy can be useful here. The analysis of training behavior is also interesting in this context. This work compares the following training strategies:

- **From Scratch**: This method trains the model from scratch. The weights are initialized randomly. Training requires a large amount of data for good results and is associated with a long training time. This method is particularly suitable for datasets similar to the ones we employed.
- **Freezing Pretrained Weights**: This method can train the pre-trained models based on a similar dataset. The earlier layers in the model are frozen so that the model can benefit from the general features, while the upper layers are adapted to the specific classes. The main advantage is that it reduces training time and improves performance.
- **Fine-tuning Pretrained Weights**: This method is particularly suitable when dealing with a small dataset with many classes, using the pre-trained weights as a baseline better to capture the differences and subtleties of the 36 classes. It balances efficiency and adaptability, offering a flexible and versatile approach to training.

In this work, ResNet50 and ResNet152 have been chosen because of their proven effectiveness in feature extraction and various classification tasks. Our models are implemented on the TensorFlow platform and trained on NVIDIA GeForce RTX 4090 GPUs (NVIDIA Corporation, Santa Clara, CA, USA) NVIDIA GeForce RTX 4090 GPUs with 24 GB of memory. The learning rate is set to 0.001, with a batch size of 16. The model is trained for 100 epochs, and early stopping with a patience parameter of 20 epochs for training and validation has been implemented.

#### Classification Metrics

To evaluate the performance of the models, the following metrics have been used:

- **Accuracy**: Accuracy measures the proportion of correctly classified instances (positive and negative) out of the total cases. The formula is given by:
  $$\mathrm{Accuracy}=\frac{TP+TN}{TP+TN+FP+FN}$$
- **Precision**: Precision indicates how many of the instances classified as positive are positive:
  $$\mathrm{Precision}=\frac{TP}{TP+FP}$$
  High precision means that the classifier produces few false positives. This metric is critical when the cost of false positives is high.
- **Recall (Sensitivity)**: Recall measures how many of the actual positive instances were correctly identified:
  $$\mathrm{Recall}=\frac{TP}{TP+FN}$$
  High recall means that the classifier produces few false negatives. This metric is essential when missing positive instances (false negatives) is costly.
- **F1 Score**: The F1 score is the harmonic mean of precision and recall (sensitivity), balancing both metrics:
  $$F1\text{-}\mathrm{Score}=2\times \frac{\mathrm{Precision}\times \mathrm{Recall}}{\mathrm{Precision}+\mathrm{Recall}}$$

Here, *TP* stands for true positive, *TN* for true negative, *FP* for false positive, and *FN* for false negative. Accuracy is helpful if the class distribution is balanced and the costs of incorrect classifications are the same for both classes. A high recall is necessary when missing positive instances (false negatives) is costly. High precision is critical when the cost of false positives is high. The F1 score is most useful when a balance between precision and recall is required, and the class distribution is unbalanced.

## 4. Dataset Characteristics

This section presents the characteristics of the dataset of the various milling tools, each of which has different geometries due to specific machining tasks that will be discussed in this section.

### 4.1. Milling Tools Geometrical Characteristics and That Application

Different types of milling cutters, such as end mills, face mills, and slitting cutters, vary in geometry and application. End mills are versatile tools for various tasks, face mills are ideal for surface milling, and slitting cutters are perfect for creating deep grooves. Each type of milling cutter is optimized to remove material more effectively for specific operations.

HSS (high-speed steel) cutters are more ductile and flexible but less complicated and wear-resistant than cutters made from carbide or ceramic. HSS is particularly well suited for softer materials and applications with lower cutting speeds. In contrast, more rigid materials, such as carbide, offer higher cutting speeds, longer tool life, and better performance when machining rigid materials.

The main difference between milling techniques lies in the material removal process. In climb milling, the cutter moves in the same direction as the material feed, resulting in a smoother surface finish and reduced tool wear but requiring the workpiece to be firmly secured. In conventional milling, the cutter moves against the material feed, which is more stable but demands more force and often produces a rougher surface.

The precision of a milling cutter is influenced by the number of cutting edges, geometry, and diameter. More cutting edges lead to a finer surface finish, while the tool’s geometry affects material removal accuracy. A smaller cutter diameter allows for more precise machining, while larger cutters operate faster but less accurately.

The key differences between the cutting tools lie in their geometry, such as the cutting angles and shapes and the number and arrangement of the edges. These factors affect material removal, cutting forces, surface quality, and suitability for various materials and applications. Depending on their design, cutters may specialize in different processes and materials, with edges being straight, spiral, or rounded. In addition, cutters can be divided into evenly spaced and unevenly spaced tools. For example, evenly spaced cutters may have edges arranged at 90°, 90°, 90°, and 90°, while unevenly spaced cutters could have edges at 90°, 45°, and 30°.

The arrangement of the cutting edges also influences the cutting process and surface quality. Evenly spaced edges result in consistent loading and a smoother finish, while unevenly spaced edges reduce vibrations and provide smoother operation. This arrangement also affects chip formation and evacuation, influencing the milling process’s efficiency and precision.

Different end-face grinds on milling cutters affect the cutting geometry and machining quality. A flat grind allows for precise and clean surface machining, while convex or concave grinds are suited for specialized tasks like form milling, impacting surface finish and chip evacuation.

The size of the chip gullet also influences chip evacuation. A larger gullet provides better chip removal and is ideal for softer materials, while a smaller gullet enhances stability, making it more suitable for more complex materials and precise cuts. The method of chip evacuation is determined by the cutter’s spiral design, which directs chips upward or downward, and by the tooth geometry, which affects chip flow and the tool’s suitability for various materials.

The clearance angles of a milling cutter impact cutting forces and tool life. A larger clearance angle reduces friction and eases the cutting of soft materials, but it may reduce stability. A smaller clearance angle is better for more complex materials but increases friction and heat.

Different edge grinds, such as pointed, rounded, or tapered, influence cutting forces, surface finish, and tool life. A pointed grind offers high sharpness for precise cuts, while a rounded grind improves stability for hard materials and is suitable for deep cuts.

Radial back clearances also affect edge sharpness and chip evacuation. A more muscular back clearance reduces friction and aids chip removal, although it is better for lighter cuts. In contrast, lower back clearance is preferable for heavier cuts, although it can increase friction.

Radial relief refers to machining the cutting edge of a milling cutter to allow for slight recession of the surface behind the cutting edge. This reduces friction between the tool and the workpiece, improves chip removal, and extends tool life.

Coated cutters feature a thin material layer such as TiN (titanium nitride) or TiAlN (titanium aluminum nitride), which enhances hardness, reduces friction, and improves the tool’s heat resistance. Uncoated cutters lack this protective layer, making them less resistant to wear and heat but often more cost-effective and sufficient for softer materials.

Coatings like TiN, TiAlN, and diamond extend tool life, reduce wear, and enhance performance for different materials and cutting speeds. These coatings differ in hardness, temperature resistance, and friction reduction. TiN provides good wear protection, TiAlN is suitable for higher temperatures, and diamond coatings offer extreme hardness but are more sensitive to impacts.

Coolants also vary in their cooling and lubrication properties. Emulsions provide excellent cooling and chip removal, cutting oils improve lubrication and surface quality, while compressed air is mainly used for chip evacuation and minimal cooling. The choice of coolant impacts tool life, cutting speed, and the quality of the machined workpiece.

### 4.2. Examples of Milling Cutters for Illustration of the Light Intensive Curves

The dataset includes the following types of milling cutters:

- **End Mills**: Versatile tools used for various operations, such as side milling, slotting, and contouring. They are available in multiple shapes like flat, ball-nose, and corner-radius.
- **Face Mills**: Designed for cutting large flat surfaces quickly. They have replaceable carbide or ceramic inserts and are ideal for surface finishing or roughing.
- **Slitting Cutters (Slitting Saws)**: Thin circular cutters are used for cutting narrow slots or for parting workpieces. These are often used in metalworking for deep cuts.
- **T-Slot Cutters**: Specifically shaped to cut T-slots into a workpiece, commonly used for machine tables to hold fixtures or clamps.
- **Ball-Nose End Mills**: Have a rounded tip helpful for 3D contouring or machining rounded shapes and cavities, especially in mold and die-making.
- **Roughing End Mills**: Feature serrated cutting edges that allow higher material removal rates and reduce cutting forces, making them ideal for heavy roughing operations.
- **Chamfer Mills**: Used to create beveled edges or chamfers on the workpiece for deburring or to prepare surfaces for welding.
- **Corner Radius End Mills**: Have rounded corners that reduce chipping and provide a smoother transition between surfaces, ideal for reducing stress concentrations in parts.
- **Shell Mills**: Large-diameter cutters that fit onto a shank, often used for heavy material removal or face milling large surfaces.
- **Keyway Cutters**: Designed to cut keyways or slots for shafts and other machine components.

#### End Mill Geometry

The dataset comprises a total of **36 different tool types**, including *taps, twist drills, end mills, face mills, T-slot cutters, deburring cutters*, and *center drills*. These tools vary not only in their geometry but also in their coatings and material properties, making the dataset unique for evaluating neural networks.

**Figure 6:**
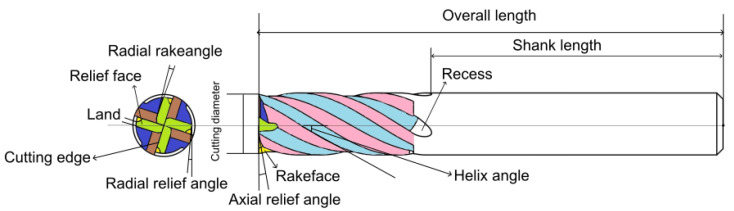
Geometry of an end mill with critical features such as rake angle, relief angle, helix angle, and cutting edge, essential for tool classification and regrinding.

Figure 6 schematically shows the most critical parameters of a milling cutter that determine the type of tool. The relevant parameters that are used as features for extracting features include

- **Cutting edge geometry**, consisting of cutting edge, flank face and rake face;
- **Angle parameters**, such as helix angle, axial and radial clearance angle;
- **Chip angle**, which influence chip formation and cutting forces;
- **Dimensions**, including cutting diameter, total length and shank length.

In addition to the pure geometry, **coatings** also influence the visual appearance and the tool’s wear and temperature properties. A detailed overview of the use and application areas of various tools can be found in Appendix A.

## 5. Results and Discussion

This study uses two distinct datasets to compare the performance of the deep learning models ResNet50 and ResNet152 on a multi-class classification task. These datasets, provided by Linner GmbH Werkzeugfabrik, were captured under different lighting conditions.

- **Dataset 1:** Tool image captured under daylight.
- **Dataset 2:** Tool image captured using a diffuse direct ring light.

### 5.1. Behavior Analysis of Metrics Curves: Loss, Accuracy, F1, Recall, and Precision

**Figure 7:**
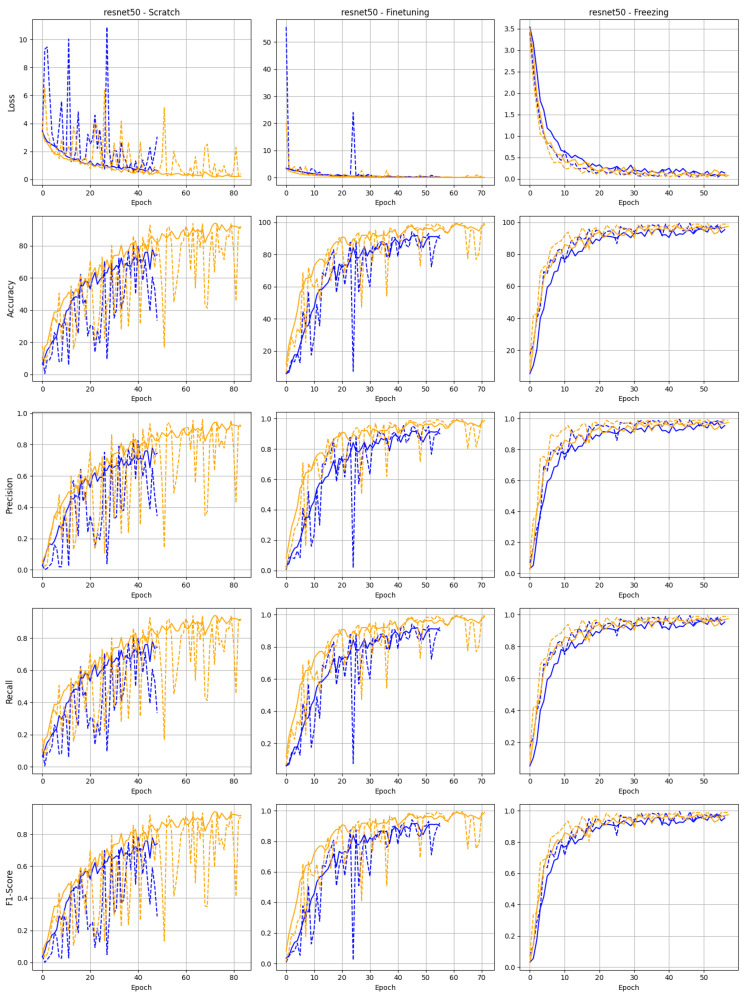
Training (continuous) and validation (dashed) curve for the ResNet50 model trained on data acquired using daylight (yellow) and diffuse ring light illumination (blue).

**Figure 8:**
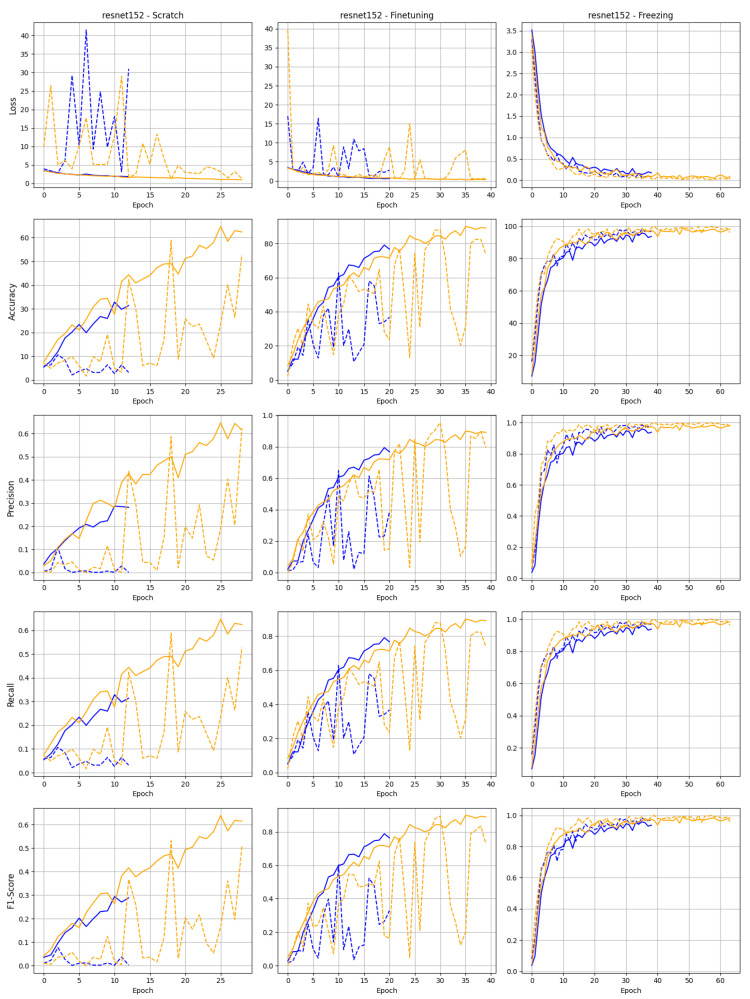
Training and validation curve for the ResNet152 model trained on data acquired using daylight (yellow) and diffuse ring light illumination (blue).

The training behavior of both datasets for the ResNet50 and ResNet152 architectures indicates that both models perform better with Dataset 2, where images were recorded under daylight conditions, compared to the dataset created using the acquisition system with ring illumination, as shown in Figure 7 and Figure 8. This suggests that the reproducibility of the images is not crucial for training the different models with varying training strategies.

It can also be seen that the curve of the remaining metrics, such as F1 and accuracy, also tends to show better results for the dataset with daylight illumination. The learning behavior of the learning validation loss curve and metric curves of various training strategies are illustrated in Figure 7 and Figure 8, starting with the training strategy from scratch. The figures show that the learning validation loss curve flattens out as expected. When trained from scratch, both models exhibit significant fluctuations in validation loss and other metrics, particularly ResNet152, which experiences sharp spikes in loss during the initial training epochs. The instability observed is likely a result of the increased depth of ResNet152, which makes it more challenging to optimize without pre-trained weights. In contrast, ResNet50 exhibits more stable training progression, even when trained from scratch. This is especially evident with the dataset containing natural daylight, where ResNet50 demonstrates faster accuracy, precision, recall, and F1 score convergence. This suggests that ResNet50 is more efficient at learning from limited data or fewer training epochs, possibly due to its lower complexity than ResNet152.

Both models display markedly smoother curves in the fine-tuning scenario, with a rapid decline in loss and a steady increase in all performance metrics. This highlights the benefit of leveraging pre-trained weights to improve convergence speed and stability, especially for deeper networks like ResNet152, which performs notably better when fine-tuned than when trained from scratch. ResNet50, while already showing strong performance from scratch, further improves under fine-tuning, reaching high levels of accuracy and precision, indicating that it adapts quickly to new data when initialized with pre-trained weights.

The freezing strategy yields the most consistent results, with both models demonstrating minimal fluctuations across all metrics. Freezing the pre-trained layers allows the models to focus on fine-tuning the final layers, resulting in very stable performance, particularly for ResNet152. This approach also shows the best performance in terms of accuracy and precision for both models, with ResNet50 outperforming ResNet152 in terms of faster convergence, although both models achieve high final scores.

The benefits of model pretraining are reflected in the loss and metrics curves. While training models from scratch can show a slow but constant improvement during the training epochs, the model with pre-trained and fine-tuned weights shows a faster increase and more stable training performance. Using pre-trained models without fine-tuning the weights for our specific image classification tasks results in significantly stable continuous curves for loss and all other metrics. This also underlines the advantage of a pre-built model, even for this specific dataset, and the significant increase in performance it brings.

### 5.2. Comparative Analysis of Training Strategies and Best-Performing Models

This study evaluated the performance of ResNet50 and ResNet152 when trained using different strategies: fine-tuning, freezing, and training from scratch. We used images captured both with and without diffuse ring light illumination. The objective was to identify the best combination of model architecture, training method, and lighting conditions for accurate image classification within the unique dataset.

#### 5.2.1. Impact of Training Strategies

Our results show that transfer learning consistently outperforms models trained from scratch in both lighting conditions, mainly when used with frozen pre-trained weights. In particular, as shown in Table 1, freezing the pre-trained weights led to the highest accuracies for both ResNet152 and ResNet50. Using the ring light, ResNet152 reached an impressive accuracy of 98.73%, closely followed by ResNet50 at 98.31%. Without the ring light and under daylight, both models still performed well, with accuracies of 98.25% for ResNet152 and 95.61% for ResNet50. This strong performance indicates that freezing the pre-trained layers allows the models to retain valuable features learned from large datasets, which proves especially useful when training data are limited.

In contrast, models trained from scratch had significantly lower performance. ResNet50 trained from scratch without the ring light achieved a decent accuracy of 93.42%, but this dropped to 75.42% with the ring light. ResNet152, when trained from scratch, struggled even more, achieving only 53.07% without the ring light and a very low 8.47% with it. These findings emphasize the difficulties of training deep neural networks from scratch with small datasets, where overfitting and poor feature learning often become significant issues.

Fine-tuning led to mixed results. ResNet50 performed well in both lighting conditions, achieving 95.34 % accuracy with the ring light and 97.81% without it. This suggests that even slight adjustments to the pre-trained weights can enhance the model’s performance when its architecture aligns well with the dataset. However, ResNet152 did not perform as well under fine-tuning, with accuracies of 67.37% with the ring light and 81.14% without it. This difference in performance may be due to ResNet152’s more significant number of trainable parameters, making it more prone to overfitting during fine-tuning if not adequately regularised. These results are also consistent with previous findings [26], which indicate that transfer learning provides a significant overall improvement over training from scratch. In particular, the ResNet152 architecture benefits the most from transfer learning. However, the situation is different for accuracy: in our work, ResNet50 achieves higher accuracy than ResNet152, in contrast to the results of [26].

#### 5.2.2. Effect of Lighting Conditions

Lighting conditions also played an important role in model performance, though the effects were nuanced. Both models benefitted slightly from diffuse ring light illumination when using the freezing strategy. The uniform lighting likely helped ensure consistency in image features, allowing the models to leverage their pre-trained knowledge more effectively. For instance, ResNet152 saw its accuracy increase from 98.25% without the ring light to 98.73% with it.

However, better performance was often seen without the ring light for models trained from scratch or fine tuned. This could be because models trained on more natural lighting variations may learn more diverse features, potentially improving their generalization. For example, ResNet50 trained from scratch performed better without the ring light, achieving an accuracy of 93.42%, compared to 75.42% with the ring light.

#### 5.2.3. Model Architecture Considerations

Another key observation is the impact of model architecture. ResNet50 consistently outperformed ResNet152 when trained from scratch or fine-tuned. Although ResNet152 is a deeper model with a greater feature extraction capacity, deeper architectures require more data to reach their full potential. Without sufficient data, they may be more susceptible to overfitting. On the other hand, ResNet152 excelled under the freezing strategy, indicating that its deeper layers of pre-trained features are highly beneficial when left unchanged.

### 5.3. Interpretation of Grad-CAM Visualizations

The Grad-CAM visualizations generated in this study offer a deeper understanding of how different convolutional neural network (CNN) architectures (ResNet50 and ResNet152) and training strategies (scratch, fine-tuning, freezing) perform under varying lighting conditions (daylight, ringlight). The heatmaps highlight the relevant areas that contribute to the model’s prediction and illustrate differences in the localization accuracy of the features relevant to the classification.

#### 5.3.1. Effect of Lighting Conditions

One of the key findings from this analysis is the significant impact that lighting conditions have on the model’s focus. Under *daylight conditions*, both ResNet50 and ResNet152 display more localized and sharply defined areas of attention. The heatmaps show that the models focus on specific parts of the tool—mainly around the top and edges. This suggests that daylight’s consistent, balanced illumination helps the models identify important features more easily.

In contrast, the *ringlight condition* introduces more diffuse attention maps. Particularly in ResNet50, the attention appears more scattered across the entire object, especially when trained from scratch or with frozen layers. This broader focus might indicate that the lighting from the ring light complicates the model’s ability to distinguish key features, leading it to distribute attention over a larger area.

#### 5.3.2. Impact of Model Architecture

**Figure 9:**
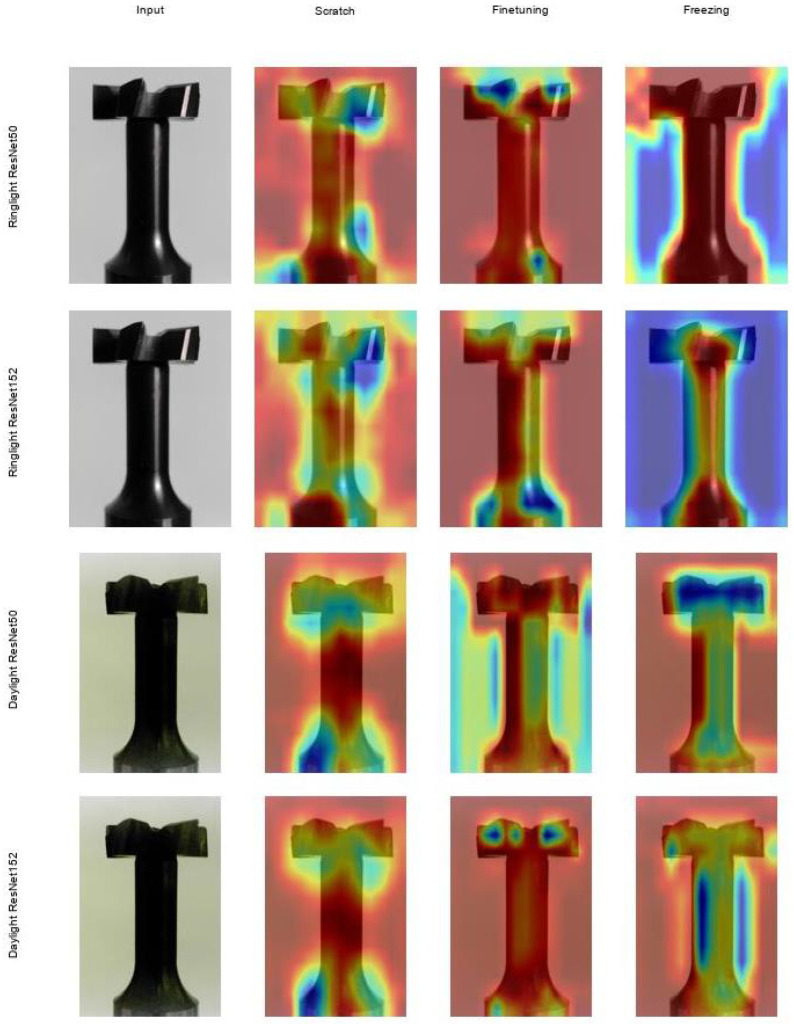
Visualization of Grad-CAM heatmaps for the classification of cutting tools (T-slot cutter) using different training strategies for the ResNet50 and ResNet152 architectures under two lighting conditions: ring light and daylight. The columns represent the training strategies: scratch, fine-tuning, and freezing. Red represents the highest activation, while blue indicates the lowest activation.

Figure 9 shows Grad-CAM heatmaps of the ResNet models for all training strategies (scratch, fine-tuning and freezing) under the two lighting conditions: Ring light and daylight illumination of a cutting tool (T-Slot Cutter). Red represents the highest activation, while blue indicates the lowest activation. The comparison between *ResNet50* and *ResNet152* reveals notable differences in how these two architectures extract and process features. *ResNet50*, being a shallower model, tends to produce more concentrated areas of attention. This is particularly noticeable in the “fine-tuning” and “freezing” strategies, where the model focuses on a strong activation (red color) almost over the entire tool surface, especially on certain parts of the object, e.g., on the upper area or along contours and edges. With the freeze training strategy, you can also clearly see the blue areas (background) around the tool in the ring lighting as opposed to the day lighting, which shows that the model pays more attention to the outer contours of the tool.

On the other hand, *ResNet152*, which has a deeper architecture, demonstrates a broader and more evenly distributed attention pattern. Its Grad-CAM visualizations show that it captures more global features, spreading attention across larger portions of the object in almost all conditions. This suggests that ResNet152’s greater depth enables it to capture more abstract and comprehensive features. While this broader attention may be beneficial for tasks that require a wider context, it could be less effective for tasks that demand highly localized feature extraction.

#### 5.3.3. Effect of Training Strategies

The choice of training strategy also significantly influences how the models direct their attention.

##### Training from Scratch

ResNet50 and ResNet152 show more expansive and less focused attention maps when trained from scratch. This behavior suggests that the models are still learning to identify essential features from the dataset and have not converged on specific regions of interest. The models explore different parts of the object, leading to more diffuse attention maps.

##### Fine-Tuning

In contrast, fine-tuning the models using pre-trained weights results in much more refined and targeted attention. The Grad-CAMs from both models show a clearer focus on the most relevant regions of the object. For example, ResNet50 highlights specific areas, such as the upper section of the object, while ResNet152 also shows a more concentrated focus across the object, though its attention remains broader compared to ResNet50.

##### Freezing Layers

Freezing earlier network layers appears to result in broader attention maps, particularly in ResNet152. This likely reflects the model’s reliance on higher-level, pre-trained features, which are more general and less specific to the new task. The Grad-CAMs suggest that the model is leveraging these generalized features to understand the object as a whole rather than focusing on fine-grained details.

### 5.4. General Observations and Implications

Overall, the Grad-CAM visualization highlights several important considerations for choosing architectures and training strategies in computer vision tasks. Lighting conditions play a major significant role in shaping model attention. Under *daylight*, models tend to have a more focused and localized attention span, while *ring light* conditions lead to broader, more diffuse attention patterns. This difference could be attributed to the uniform lighting in daylight, which allows the model to detect key features more quickly, whereas the harsher, uneven lighting in ring light complicates feature detection.

The comparison between *ResNet50* and *ResNet152* suggests that deeper architectures (like ResNet152) capture more global context, while shallower architectures (like ResNet50) tend to concentrate on more specific regions. It is likely that deeper models are better suited for tasks where a broader understanding of the object is required, while shallower models may excel in tasks that depend on pinpointing detailed features.

Finally, the choice of *training strategy* significantly affects how attention is distributed. *Fine-tuning* is the most effective strategy in producing precise attention maps, indicating that it helps models efficiently leverage pre-trained features to focus on the most relevant areas. In contrast, training from scratch encourages more exploratory attention behavior, and *freezing* layers result in a broader, less focused attention span, which may still benefit tasks requiring a broader perspective.

In conclusion, this Grad-CAM analysis underscores the importance of understanding how different factors, such as lighting conditions, model architecture, and training strategy, influence how deep learning models process visual data. The findings suggest that *daylight* conditions and *fine-tuning* are optimal for tasks requiring precise feature extraction, while deeper models like *ResNet152* and training strategies like *freezing layers* may be better suited for tasks that benefit from a more global understanding of the object. These insights could help guide future model selection and training strategies in various computer vision applications, mainly when lighting conditions or task requirements vary.

## 6. Conclusions

In this work, an AI-based application for tool classification for machine repair was developed and explored. Different training strategies were validated with two specific datasets of tools taken under various illumination conditions: direct ring lighting and daylight. For the analysis, the state-of-the-art ResNet50 architecture and its extended version, ResNet152, were used. The specific training dataset for the AI model consisted of 1728 images of 36 different complex-shaped tool types.

In this study, different models trained with various training strategies, including training from scratch, using pre-trained models, and freezing pre-trained weights, were compared. The results indicated that the fine-tuning training strategy performed best. When comparing the effect of lighting conditions, Dataset 2, acquired with ring illumination, achieved the highest accuracy: 0.987 for ResNet152 and 0.983 for ResNet50 when freezing trainable parameters. However, models trained on Dataset 1 (daylight images) tended to perform better in terms of both training strategies, fine-tuning and scratch, than those acquired with ring illumination.

By analyzing the feature maps, this study showed that CNN architectures cannot extract apparent features, such as edges, using daylight illumination when trained from scratch. Grad-CAM analysis demonstrated that the feature extraction of the different tools on the different layers strongly depends on the type of lighting. For example, in the data acquired with ring lighting, the model could extract information from the tool edges in all training strategies. At the same time, feature extraction in daylight images tended to focus on finer textures, such as surface roughness.

It was clearly shown that pre-trained models with ImageNet outperform other training strategies on both datasets and provide a solid basis for AI applications in industrial settings. In future work, the classification of more than 300 tool types and a benchmark analysis of state-of-the-art CNN architectures are planned. These classifiers will be designed for industrial environments and aim to classify tools that have never been seen before.

However, there are some limitations. There are no other datasets from other tools in the production environment that belong to the same classes but differ fundamentally in terms of different color representations or damage, such as chipping on the edges caused by wear or lubricating material. In addition, the tools were recorded in daylight at the same time of day, which means that variations in daylight, depending on the time of day, also affect the model’s performance. Therefore, in the future, we will generate more datasets of tools from the production environment to investigate these limitations.

## 7. Implications and Future Work

These results highlight the effectiveness of transfer learning, especially the freezing strategy when working with limited data. Pre-trained models significantly reduce the need for large datasets and intensive computational resources while providing high accuracy. The mixed effects of lighting conditions suggest that while controlled lighting can improve model performance under specific training strategies, natural light variations can help models learn a wider range of features.

For practitioners, these results emphasize the importance of selecting appropriate training strategies and carefully considering the conditions under which images are acquired. The use of pre-trained models with frozen weights is a robust and reliable approach, especially if consistent illumination can be ensured. What has not yet been investigated in this work is how the classification worked with nine unknown tools. Here, tools of different categories can also have different surface properties such as coating or roughness, and they vary in size. Therefore, the robustness of the dataset will be increased in the future so that a comprehensive statement about the usability of the classifier in real production conditions can be evaluated. In the future, it will be valuable to investigate if these results can be generalized to other datasets and image classification tasks. In addition, exploring the impact of data augmentation and regularization techniques could help to further optimize model performance, especially when training data are limited. Furthermore, various state-of-the-art neural networks will be compared and the different training strategies, especially from scratch, will be evaluated to draw conclusions about their learning behavior and efficiency.

## Figures and Tables

**Table 1:** Comparison of accuracy and weighted metrics under different lighting conditions.

Condition	Experiment	Model	Accuracy	Precision	Recall	F1 Score
**Dataset 2**	Fine-tuning	ResNet152	0.6737	0.7070	0.6737	0.6320
		ResNet50	0.9534	0.9600	0.9534	0.9525
	Freezing	ResNet152	0.9873	0.9876	0.9873	0.9872
		ResNet50	0.9831	0.9838	0.9831	0.9830
	Scratch	ResNet152	0.0847	0.0403	0.0847	0.0495
		ResNet50	0.7542	0.7897	0.7542	0.7367
**Dataset 1**	Fine-tuning	ResNet152	0.8114	0.8419	0.8114	0.7993
		ResNet50	0.9781	0.9849	0.9781	0.9785
	Freezing	ResNet152	0.9825	0.9850	0.9825	0.9822
		ResNet50	0.9561	0.9624	0.9561	0.9525
	Scratch	ResNet152	0.5307	0.5127	0.5307	0.4647
		ResNet50	0.9342	0.9476	0.9342	0.9275

**Figure A1:**
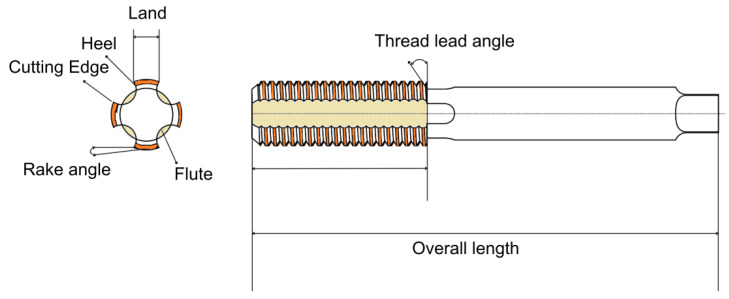
Geometry of a tapping tool with features such as rake angle, cutting edge, land, flute, and thread lead angle.

**Figure A2:**
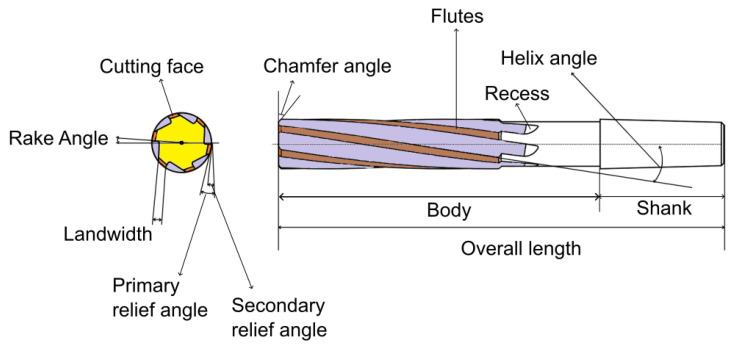
Geometry of a reamer with features such as rake angle, cutting face, chamfer angle, land width, primary and secondary relief angles, flutes, and helix angle.

**Figure A3:**
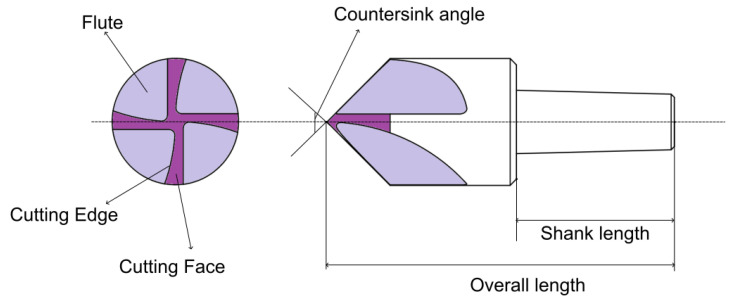
Geometry of a countersink with features such as cutting edge, cutting face, flute, countersink angle, overall length, and shank length.

**Figure A4:**
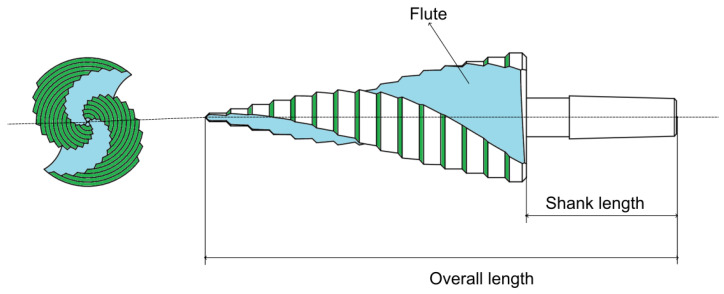
Geometry of a step drill bit with features such as flutes, overall length, and shank length.

## Data Availability

The data supporting the findings of this study are owned by WMH Herion Antriebstechnik GmbH, Stanglmühle 9-11, 85283 Wolnzach, Germany, and are not publicly available due to proprietary restrictions. However, data may be available from the authors upon reasonable request and with permission of WMH Herion Antriebstechnik GmbH.

## Abbreviations

The following abbreviations are used in this manuscript:

CNC	Multidisciplinary Digital Publishing Institute
CNN	Convolutional neural network
DTL	Digital Tool Intelligence
TiN	Titanium nitride
TiAlN	Titanium aluminum nitride
MCC	Multi-class classification

## Appendix A. Tool Geometries

### Appendix A.1. Tapping Tool Geometry

A tapping tool has several key geometrical features that make it practical for cutting internal threads. It has flutes running along its length to evacuate chips and allow coolant to reach the cutting edges. The thread form matches the desired internal thread profile, and the chamfer at the front of the tap gradually cuts threads. The pitch diameter ensures the fit of the cut thread, while the land supports the cutting edges. The helix angle influences chip removal, with steeper angles better suited for softer materials. The tool’s shank allows it to be clamped, and the core diameter provides strength and rigidity. The overall length determines how deep the tap can cut.

### Appendix A.2. Reamer Geometry

A reamer drill bit is designed for precision finishing and is used to enlarge and smooth out pre-drilled holes to exact dimensions. It has several key geometrical features that contribute to its function. The cutting edges, known as flutes, are straight or spiral and help to remove small amounts of material while ensuring a smooth finish. These flutes also allow chips to be evacuated from the hole during reaming. The diameter of the reamer is carefully controlled to ensure accurate hole sizing, and the number of flutes varies, with more flutes providing a finer finish.

The reamer’s chamfer or tapered lead helps guide the tool into the hole for smooth engagement and gradual material removal. The land between the flutes supports the cutting edges and helps stabilize the tool. The shank allows the reamer to be held in a machine or hand tool, while the core diameter offers rigidity and strength to the tool. In general, the reamer is designed for precision and accuracy, ensuring that the holes are perfectly sized and have a smooth finish.

### Appendix A.3. Countersink Geometry

A countersink tool is used to create a conical recess around a hole to allow the head of a screw or bolt to sit flush with or below the surface. Its geometry includes a conical cutting edge with a specific angle, typically between 60° and 90°, which determines the depth and shape of the recess. The tool has multiple cutting edges, often arranged in a spiral or helical pattern, to remove material and efficiently produce a clean, smooth finish. The shank of the countersink tool fits into a drill or tool holder, while the overall design allows it to create precise tapered openings for fasteners.

### Appendix A.4. Drilling Tool Geometries

A drilling bit is designed with specific geometric features to create holes in various materials effectively. The tip of the bit has a pointed end, typically angled between 118° and 135°, which helps to initiate the drilling process and penetrate the material. The cutting edges at this point are precisely ground to enhance cutting efficiency.

The bit features helical flutes that spiral along its length. These flutes are essential for removing material as it is drilled and for allowing chips to escape. The shape and number of flutes significantly affect how effectively the bit clears chips and maintains smooth drilling. The angle of these flutes, known as the helix angle, also impacts chip removal and overall drilling performance; a steeper angle lifts chips out more efficiently.

The body of the bit, extending from the tip to the shank, is often tapered to reduce friction and enhance efficiency. The cylindrical shank fits into the drill or tool holder, providing support and alignment during drilling. The bit’s diameter determines the hole’s size: larger diameters are suited for making bigger holes, while smaller diameters are ideal for precision work. Together, these features ensure the drilling bit can cut through various materials effectively and be tailored to meet different drilling tasks.

### Appendix A.5. Step Drill Bit Geometry

A step drill bit, also known as a “uni-bit,” is designed for creating holes of varying diameters in a single pass. Its geometry features a tapered, cone-like shape with multiple stepped diameters. Each step is different, allowing the bit to drill holes of various sizes without switching tools.

The cutting edges of a step drill are arranged in a spiral or helical pattern around the bit’s cone, which efficiently removes material and reduces friction. The gradual increase in diameter from the tip to the larger end helps the bit drill progressively larger holes. This design allows drilling multiple hole sizes and provides a clean, smooth finish.

The step drill bit’s shank is cylindrical, enabling it to be securely held in a drill or tool holder. The overall shape of the bit, with its conical and stepped design, allows for versatility in drilling tasks, making it especially useful for working with sheet metal, plastic, and other materials requiring multiple hole sizes. The step drill bit eliminates the need for various drill bits, simplifying the drilling process and improving efficiency.
